# Acupuncture decreased the risk of stroke among patients with fibromyalgia in Taiwan: A nationwide matched cohort study

**DOI:** 10.1371/journal.pone.0239703

**Published:** 2020-10-01

**Authors:** Ming-Cheng Huang, Hung-Rong Yen, Cheng-Li Lin, Yu-Chen Lee, Mao-Feng Sun, Mei-Yao Wu

**Affiliations:** 1 Department of Chinese Medicine, China Medical University Hospital, Taichung, Taiwan; 2 School of Chinese Medicine, College of Chinese Medicine, Graduate Institute of Chinese Medicine, China Medical University, Taichung, Taiwan; 3 Department of Medical Research, China Medical University Hospital, Research Center for Traditional Chinese Medicine, Taichung, Taiwan; 4 Chinese Medicine Research Center, China Medical University, Taichung, Taiwan; 5 Department of Biotechnology, Asia University, Taichung, Taiwan; 6 Management Office of Health Data, China Medical University Hospital, Taichung, Taiwan; 7 Graduate Institute of Acupuncture Science, College of Chinese Medicine, China Medical University, Taichung, Taiwan; 8 School of Chinese Medicine, College of Chinese Medicine, China Medical University, Taichung, Taiwan; 9 School of Post-Baccalaureate Chinese Medicine, College of Chinese Medicine, China Medical University, Taichung, Taiwan; Humanitas Clinical and Research Center - IRRCS, ITALY

## Abstract

**Background:**

The aim of this study was to investigate the effectiveness of acupuncture in decreasing the risk of stroke in patients with fibromyalgia.

**Methods:**

We enrolled patients who was newly diagnosed as having fibromyalgia between 1 January, 2000 and 31 December, 2010 from the Taiwanese National Health Insurance Research Database. The claim data for both the acupuncture cohort and non-acupuncture cohort were assessed from the index date to 31 December, 2013. A Cox regression model adjusted for age, sex, comorbidities, and drugs use was used to compare the hazard ratios of the two cohorts. The cumulative incidence of stroke was estimated by using the Kaplan–Meier method.

**Results:**

After performing a propensity score matching with a 1:1 ratio, there were 65,487 patients in the acupuncture and non-acupuncture cohorts with similar distributions in the baseline characteristics. The cumulative incidence of stroke was significantly lower in the acupuncture cohort (log-rank test, p < 0.001). In the follow-up period, 4,216 patients in the acupuncture cohort (11.01 per 1000 person-years) and 6,849 patients in the non-acupuncture cohort (19.82 per 1000 person-years) suffered from stroke (adjusted HR 0.53, 95% CI 0.51–0.55). Acupuncture favorably affected the incidence of stroke regardless of the patient’s age, sex, comorbidities, and conventional drug use.

**Conclusions:**

Our study found that acupuncture might have a beneficial effect on reducing the risk of stroke in patients with fibromyalgia in Taiwan. Additional clinical and basic science studies are warranted.

## Introduction

Fibromyalgia is an autoimmune disease with a prevalence of 1~5% in the general population worldwide [1]. It is reported that females are 1.64 times more likely than males to develop fibromyalgia. One recent meta-analysis showed even larger gender difference, with the prevalence of 3.98% for female and 0.01% for male [2]. Clinical manifestations of fibromyalgia include widespread pain for more than three months, accompanied by headache, impaired daily activities, and emotional distresses [3]. Patients with fibromyalgia tend to have comorbidities of depression, insomnia, migraine, irritable bowel syndrome, systemic lupus erythematosus, and rheumatoid arthritis [4]. The pathogenesis of fibromyalgia has remained unclear up to now. The possible mechanism is central sensitization, in which dysfunction of central nervous system involving ascending and descending neural pathways leads to an amplified response to stimulation [5].

Therapeutic recommendations for fibromyalgia include pregabalin and duloxetine. Patients suffering from adverse effects of these drugs are prone to use nonpharmacologic treatments, such as exercise, massage and acupuncture [3, 5]. Previous study revealed that one in five of fibromyalgia patients received acupuncture within the first two years of initial diagnosis [6], because it’s an effective and safe treatment for relieving pain, reducing anxiety, and mitigating insomnia in patients with fibromyalgia [7].

One population-based study showed that patients with fibromyalgia, particularly younger patients, had a higher risk of stroke than those without fibromyalgia [8]. Comorbidities of fibromyalgia, which include depression and sleep disorders, were also associated with a higher risk of stroke [9, 10]. Some clinical trials have demonstrated the efficacy of acupuncture treatment in patients with fibromyalgia [11–17]. However, there was no long-term follow-up study to evaluate whether acupuncture could prevent stroke in patients with fibromyalgia.

In Taiwan, the National Health Insurance (NHI) program was established in 1995 by the National Health Insurance Administration (NHIA) and provided coverage to more than 23.03 million residents in Taiwan. Traditional Chinese medicine (TCM) services, including acupuncture, have been reimbursed since 1996. The Taiwanese National Health Insurance Research Database (NHIRD) includes demographic, diagnostic, interventional and long-term follow-up data on more than 99% of the population in Taiwan [18]. To determine whether acupuncture could decrease the risk of stroke in patients with fibromyalgia, we used the NHIRD to conduct a population-based cohort study.

## Materials and methods

### Data sources

We used the Longitudinal Health Insurance Database 2000 (LHID 2000), which contains medicine-related information on 1 million beneficiaries randomly sampled from the registry of all beneficiaries in 2000. The sampled patients exhibited no significant differences in age, sex, birth year, or average insured payroll-related amount in comparing with general population. The International Classification of Diseases, Ninth Revision, Clinical Modification (ICD-9-CM) codes were used for diagnoses. Because the NHIRD contains secondary data identified for research, the requirement for informed consent was waived for the present study. This study was approved by the Institutional Review Board of China Medical University Hospital (CMUH104-REC2-115).

### Study population

**Fig 1:**
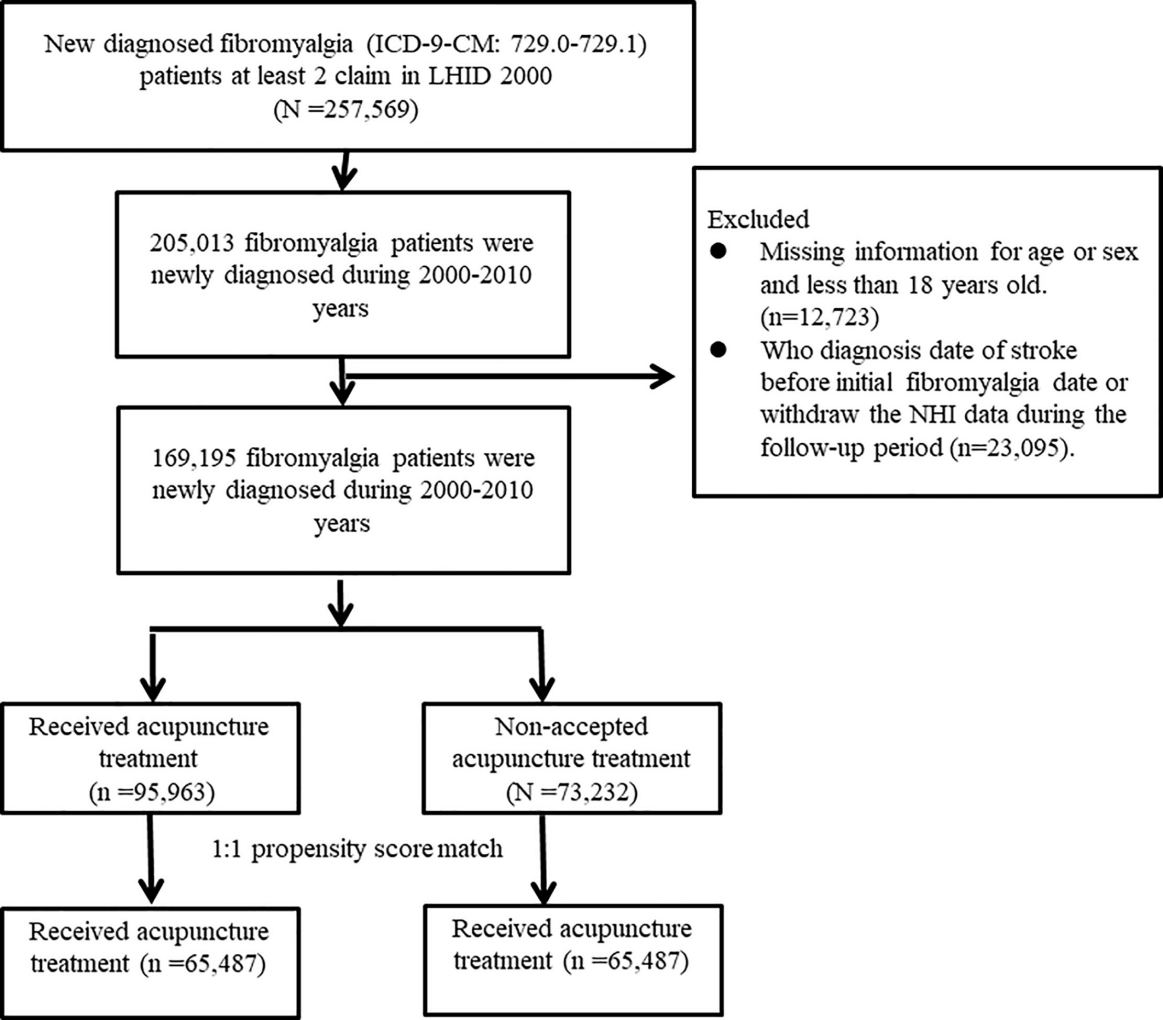
Study population flowchart. We identified 169,195 patients who were newly diagnosed with fibromyalgia between 2000 and 2010. After 1:1 propensity score matching by sex, age, comorbidities, drug use, diagnostic year and index year was performed, the acupuncture cohort and non-acupuncture cohort both comprised 65,487 patients.

We identified the patients who were newly diagnosed as having fibromyalgia with at least two ambulatory claims or one in-patient claim with ICD-9-CM diagnostic codes of 729.0 or 729.1 from 2000 to 2010 (Fig 1). Patients with fibromyalgia who received acupuncture after the initial diagnosis of fibromyalgia were enrolled in the acupuncture group, where patients who never received acupuncture between the initial diagnosis of fibromyalgia and the end of follow-up were enrolled in the non-acupuncture group. The index date was the first date that patients started to receive acupuncture after the initial diagnosis of fibromyalgia, and it was randomly assigned for the non-acupuncture group. The claim data for both cohorts were assessed from the index date to 31 December, 2013 (end of the study), the time of diagnosis of stroke, or when the patients were censored for withdrawal from insurance or were lost to follow-up.

### Covariate assessment

The sociodemographic factors included patient’s age and sex. The patient’s age were divided into 3 groups: 18–39 years old, 40–64 years old and ≥65 years old. Baseline comorbidities were considered present if ICD-9-CM codes appeared at least two times in the outpatient claims or at least one time in the inpatient claims before the initial diagnosis of fibromyalgia. Comorbidities analyzed in the current study included diabetes mellitus (ICD-9-CM: 250), hypertension (ICD-9-CM: 401–405), hyperlipidemia (ICD-9-CM: 272), coronary artery disease (ICD-9-CM: 410–413, 414.01–414.05, 414.8, 414.9), atrial fibrillation (ICD-9-CM: 427.3, 427.31, 427.32), irritable bowel syndrome (ICD-9-CM: 564.1), interstitial cystitis (ICD-9-CM: 595.1), alcoholism (ICD-9-CM: 291, 303, 305.00, 305.01, 305.02, 305.03, 790.3 and V11.3), tobacco dependence (ICD-9-CM: 305.1) and obesity (ICD-9-CM: 278 and A183). The use of NSAIDs, oral steroids, antidepressants, and statins were also adjusted in the model.

### Statistical analysis

We used 1:1 propensity score matching by sex, age (per 5 years), baseline comorbidities (including diabetes mellitus, hypertension, hyperlipidemia, coronary artery disease, atrial fibrillation, irritable bowel syndrome, interstitial cystitis, alcoholism, tobacco dependence, obesity), drug use (including NSAIDs, oral steroids, antidepressants, and statins), diagnostic year of fibromyalgia and index year to reduce the influence of confounding factors.

We used the standardized mean difference to compare the differences in the baseline characteristics between the acupuncture and non-acupuncture cohorts. Standardized mean differences less than 0.1 standard deviation (S.D.) indicated a negligible difference between the two groups. The hazard ratio (HR) and 95% confidence interval (95% CI) were calculated for each variable by Cox proportional hazard regression. The difference in the prevalence of stroke between the two cohorts was estimated using the Kaplan–Meier method and the log-rank test.

Both the statistical analyses were performed and the figures were created using SAS 9.4 (SAS Institute, Cary, NC) and R software. P < 0.05 for the two-tailed tests indicate statistical significance.

## Results

Table 1 shows the baseline characteristics of the acupuncture and non-acupuncture cohorts. After 1:1 propensity-score matching was performed, there were 65,487 patients in both cohorts, which had similar distributions of sex, age, baseline comorbidities and drug use. The proportion of females was higher than that of males, and 50% of patients were between 40 and 64-year-old.

**Table 1:** Characteristics of fibromyalgia patients according to whether they received acupuncture or not.

Variable	Fibromyalgia patients				Standardized mean difference
	Acupuncture				
	No (n = 65487)		Yes (n = 65487)		
	n	%	n	%	
**Sex**					
Female	36053	55.05	36194	55.27	0.004
Male	29434	44.95	29293	44.73	0.004
**Age group**					
18–39	25353	38.71	24464	37.36	0.028
40–64	32150	49.09	34307	52.39	0.066
≥65	7984	12.19	6716	10.26	0.061
Mean (SD, years)	45.42 (15.25)		45.17 (14.47)		0.017
**Baseline comorbidity**					
Diabetes mellitus	7682	11.7	7528	11.5	0.007
Hypertension	15475	23.6	15141	23.1	0.012
Hyperlipidemia	12718	19.4	12570	19.2	0.006
Coronary artery disease	5270	8.05	5144	7.85	0.007
Atrial fibrillation	270	0.41	264	0.40	0.001
Irritable bowel syndrome	4122	6.29	4086	6.24	0.002
Interstitial cystitis	63	0.10	62	0.09	0.001
Alcoholism	373	0.57	364	0.56	0.002
Tobacco dependence	380	0.58	367	0.56	0.003
Obesity	533	0.81	520	0.79	0.002
**Drug use**					
NSAIDs	64788	98.9	64819	99.0	0.005
Oral steroids	47812	73.0	47863	73.1	0.002
Antidepressants	22847	34.9	22657	34.6	0.006
Statins	12753	19.5	12629	19.3	0.005
**Types of acupuncture**					
Only manual acupuncture	-	-	56388	86.1	
Only electroacupuncture	-	-	1879	2.87	
Both	-	-	7220	11.0	
**Acupuncture visits, mean (median)**			8.73 (4)		
**Follow up years, mean (median)**	5.28 (4.82)		5.85 (5.37)		

Table 2 displays Cox proportional hazard models for the cohort with acupuncture treatment and the covariates of stroke. The risk of stroke was lower in the acupuncture cohort than that in the non-acupuncture cohort (adjusted HR: 0.53, 95% CI: 0.51–0.55). Risk factors of stroke for patients with fibromyalgia included male sex, elder age, diabetes mellitus, hypertension, hyperlipidemia, coronary artery disease, and atrial fibrillation. Patients who took NSAIDs, oral steroids or statins all had a lower risk of stroke.

**Table 2:** Cox model with hazard ratios and 95% confidence intervals of stroke according to whether the patients underwent acupuncture and other covariates among the fibromyalgia patients.

Variable	No. of event (n = 11065)	Crude*			Adjusted^†^		
		HR	(95%CI)	p-value	HR	(95%CI)	p-value
**Acupuncture**							
No	6849	1.00	reference	<0.0001	1.00	reference	<0.0001
Yes	4216	0.57	(0.55, 0.59)		0.53	(0.51, 0.55)	
**Sex**							
Female	6093	1.00	reference	0.69	1.00	reference	<0.0001
Male	4972	1.01	(0.97, 1.05)		1.13	(1.09, 1.17)	
**Age group**							
18–39	922	1.00	reference		1.00	reference	
40–64	6334	5.23	(4.88, 5.60)	<0.0001	4.23	(3.94, 4.54)	<0.0001
≥65	3809	16.7	(15.6, 18.0)	<0.0001	9.29	(8.58, 10.1)	<0.0001
**Baseline comorbidity (ref = non-site comorbidity)**							
Diabetes mellitus	2854	2.92	(2.80, 3.05)	<0.0001	1.36	(1.29, 1.42)	<0.0001
Hypertension	5849	4.04	(3.90, 4.20)	<0.0001	1.97	(1.88, 2.06)	<0.0001
Hyperlipidemia	3969	2.53	(2.43, 2.63)	<0.0001	1.29	(1.23, 1.35)	<0.0001
Coronary artery disease	2094	3.08	(2.94, 3.23)	<0.0001	1.16	(1.10, 1.22)	<0.0001
Atrial fibrillation	144	3.95	(3.36, 4.66)	<0.0001	1.36	(1.15, 1.60)	<0.0001
Irritable bowel syndrome	983	1.57	(1.47, 1.68)	<0.0001	0.97	(0.91, 1.04)	0.41
Interstitial cystitis	10	1.00	(0.54, 1.86)	0.99	0.67	(0.36, 1.25)	0.21
Alcoholism	70	1.29	(1.02, 1.63)	0.04	1.19	(0.94, 1.51)	0.14
Tobacco dependence	28	0.54	(0.38, 0.79)	0.001	0.57	(0.39, 0.82)	0.003
Obesity	75	0.89	(0.71, 1.12)	0.31	0.69	(0.55, 0.86)	0.001
**Drug use**							
NSAIDs	10655	0.18	(0.16, 0.20)	<0.0001	0.17	(0.15, 0.19)	<0.0001
Oral steroids	7390	0.65	(0.63, 0.68)	<0.0001	0.48	(0.46, 0.50)	<0.0001
Antidepressants	6739	3.05	(2.93, 3.17)	<0.0001	2.31	(2.22, 2.41)	<0.0001
Statins	2701	1.28	(1.23, 1.34)	<0.0001	0.56	(0.54, 0.59)	<0.0001

Crude HR* represented relative hazard ratio.

Adjusted HR^†^ represented adjusted hazard ratio: mutually adjusted for age, sex diabetes mellitus, hypertension, hyperlipidemia, coronary artery disease, atrial fibrillation, irritable bowel syndrome, interstitial cystitis, alcoholism, tobacco dependence, obesity and drug use (including NSAIDs, oral steroids, antidepressants, and statins) in Cox proportional hazard regression.

The beneficial effect of acupuncture on reducing the incidence of stroke was independent of sex and age. In individuals with and without diabetes mellitus, hypertension, hyperlipidemia, coronary artery disease, atrial fibrillation, irritable bowel syndrome, alcoholism, tobacco dependence and obesity, patients in the acupuncture group had a lower risk of stroke than those in the non-acupuncture group (Table 3).

**Table 3:** Incidence rates, hazard ratio and confidence intervals of stroke for fibromyalgia patients who did and did not undergo acupuncture in the stratification of sex, age, comorbidities, and drugs use.

Variables	Acupuncture treatment							
	No			Yes			Crude HR (95%CI)	Adjusted HR (95%CI)
	(N = 65487)			(N = 65487)				
	Event	Person years	IR^†^	Event	Person years	IR^†^		
**Total**	6849	345624	19.82	4216	382954	11.01	0.57(0.55, 0.59)***	0.53(0.51, 0.55)***
**Sex**								
Female	3768	191425	19.68	2325	211243	11.01	0.57(0.54, 0.60)***	0.53(0.50, 0.55)***
Male	3081	154199	19.98	1891	171711	11.01	0.56(0.53, 0.60)***	0.53(0.50, 0.57)***
**Age group**								
18–39	594	142519	4.17	328	142751	2.30	0.55(0.48, 0.63)***	0.53(0.46, 0.60)***
40–64	3850	170529	22.58	2484	204548	12.14	0.55(0.52, 0.58)***	0.53(0.50, 0.55)***
≥65	2405	32576	73.83	1404	35655	39.38	0.56(0.52, 0.60)***	0.53(0.50, 0.57)***
**Baseline comorbidity**								
Diabetes mellitus								
No	5092	311188	16.36	3119	341165	9.14	0.57(0.54, 0.59)***	0.53(0.51, 0.56)***
Yes	1757	34436	51.02	1097	41789	26.25	0.54(0.50, 0.59)***	0.52(0.48, 0.56)***
Hypertension								
No	3224	274582	11.74	1992	297888	6.69	0.57(0.54, 0.61)***	0.54(0.51, 0.57)***
Yes	3625	71042	51.03	2224	85066	26.14	0.54(0.51, 0.56)***	0.52(0.49, 0.55)***
Hyperlipidemia								
No	4421	285224	15.50	2675	313292	8.54	0.56(0.53, 0.58)***	0.52(0.50, 0.55)***
Yes	2428	60400	40.20	1541	69662	22.12	0.57(0.54, 0.61)***	0.55(0.51, 0.58)***
Coronary artery disease								
No	5559	322813	17.22	3412	355743	9.59	0.57(0.54, 0.59)***	0.53(0.51, 0.55)***
Yes	1290	22810	56.55	804	27211	29.55	0.55(0.50, 0.60)***	0.53(0.49, 0.58)***
Atrial fibrillation								
No	6760	344681	19.61	4161	381579	10.90	0.57(0.55, 0.59)***	0.53(0.51, 0.55)***
Yes	89	943	94.34	55	1374	40.02	0.48(0.34, 0.67)***	0.47(0.33, 0.68)***
Irritable bowel syndrome								
No	6242	325851	19.16	3840	361068	10.64	0.57(0.54, 0.59)***	0.53(0.51, 0.55)***
Yes	607	19773	30.70	376	21886	17.18	0.57(0.50, 0.65)***	0.55(0.48, 0.62)***
Interstitial cystitis								
No	6839	345361	19.80	4216	382569	11.02	0.57(0.55, 0.59)***	0.53(0.51, 0.55)***
Yes	10	263	37.97	0	385	0.00	-	
Alcoholism								
No	6811	343970	19.80	4184	381150	10.98	0.57(0.55, 0.59)***	0.53(0.51, 0.55)***
Yes	38	1654	22.97	32	1804	17.74	0.78(0.48, 1.25)	0.63(0.38, 1.02)
Tobacco dependence								
No	6828	343995	19.85	4209	381474	11.03	0.57(0.55, 0.59)***	0.57(0.55, 0.59)***
Yes	21	1629	12.89	7	1480	4.73	0.37(0.16, 0.87)*	0.37(0.16, 0.87)*
Obesity								
No	6804	342996	19.84	4186	380147	11.01	0.57(0.55, 0.59)***	0.57(0.55, 0.59)***
Yes	45	2628	17.1	30	2807	10.69	0.60(0.38, 0.96)*	0.60(0.38, 0.96)*
**Drug use**								
NSAIDs								
No	283	1704	166.05	127	2926	43.41	0.34(0.28, 0.42)***	0.34(0.28, 0.42)***
Yes	6566	343920	19.09	4089	380028	10.76	0.57(0.55, 0.60)***	0.57(0.55, 0.60)***
Oral steroids								
No	2407	81465	29.55	1268	92921	13.65	0.49(0.46, 0.53)***	0.49(0.46, 0.53)***
Yes	4442	264159	16.82	2948	290032	10.16	0.61(0.58, 0.64)***	0.61(0.58, 0.64)***
Antidepressants								
No	2697	231430	11.65	1629	251443	6.48	0.56(0.53, 0.60)***	0.56(0.53, 0.60)***
Yes	4152	114194	36.36	2587	131511	19.67	0.56(0.53, 0.59)***	0.56(0.53, 0.59)***
Statins								
No	5229	274018	19.08	3135	306821	10.22	0.55(0.53, 0.58)***	0.55(0.53, 0.58)***
Yes	1620	71606	22.62	1081	76133	14.20	0.62(0.58, 0.67)***	0.62(0.58, 0.67)***

Abbreviation: IR, incidence rates, per 1,000 person-years; HR, hazard ratio; CI, confidence interval.

Adjusted HR: adjusted for age, sex, diabetes mellitus, hypertension, hyperlipidemia, coronary artery disease, atrial fibrillation, irritable bowel syndrome, interstitial cystitis, alcoholism, tobacco dependence, obesity and drug use (including NSAIDs, oral steroids, antidepressants and statins) in Cox proportional hazards regression.

*:<0.05; **:<0.01

*** p<0.001.

**Fig 2:**
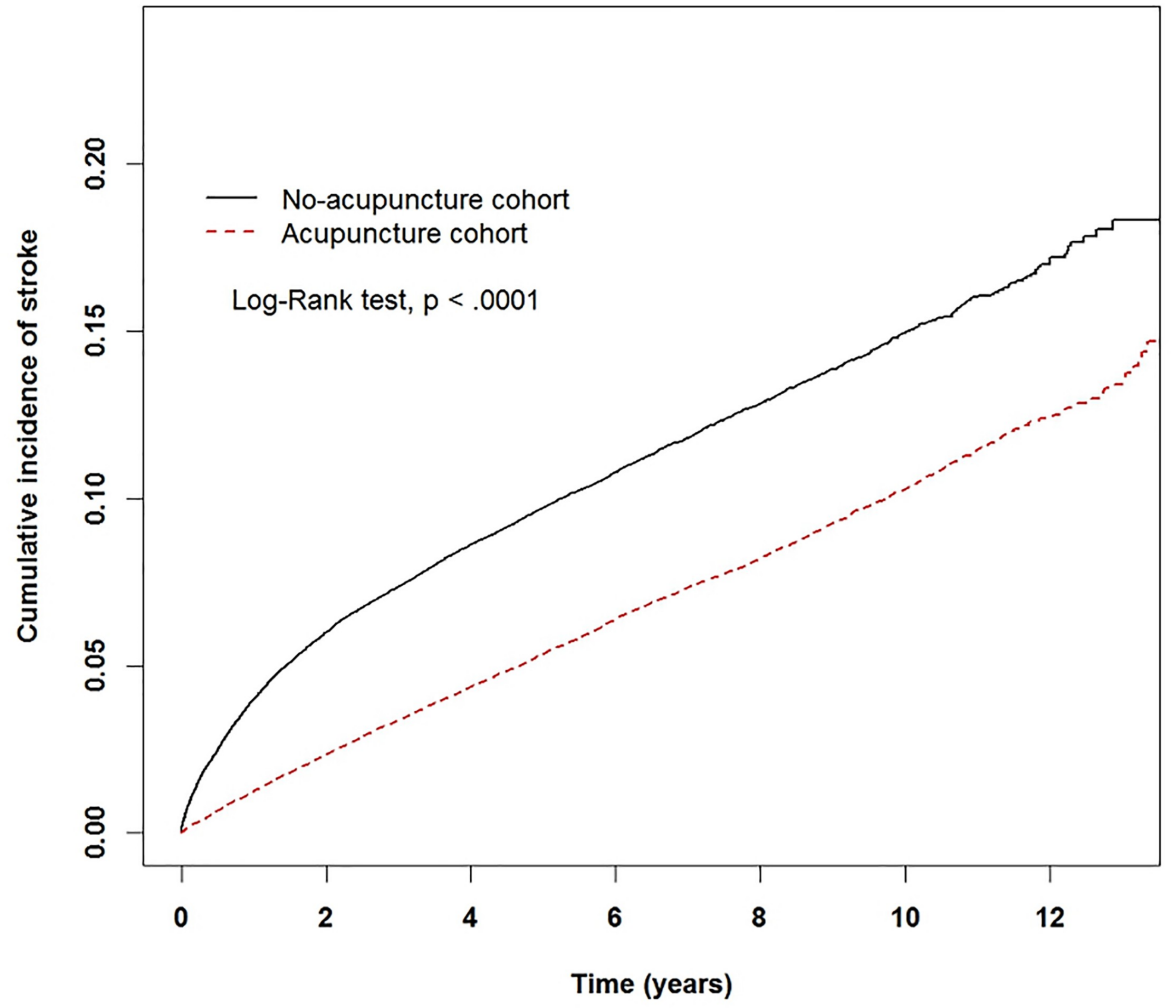
The estimated cumulative incidence of stroke for the acupuncture and non-acupuncture cohorts with fibromyalgia. Result of Kaplan-Meier analysis showed that the incidence of stroke in the acupuncture cohort was significantly lower than that in the non-acupuncture cohort.

The Kaplan-Meier analysis with the log-rank test showed a lower cumulative incidence of stroke in the acupuncture cohort than that in the non-acupuncture cohort (p<0.0001) (Fig 2).

We further analyzed the frequency distributions of the clinical visits of the patients who received acupuncture by major disease categories (as the major reason for visiting doctors) (Table 4). Patients with fibromyalgia most commonly received acupuncture for disorders of musculoskeletal and connective tissue, followed by injuries, and symptoms and signs related to neuropsychiatry disorders.

**Table 4:** The distribution of acupuncture cohort by disease categories/diagnosis in patients with fibromyalgia.

Disease (ICD-9-CM)	Acupuncture users	
	(n = 65487)	
	n	%
Musculoskeletal system and connective tissue (710–739)	48656	74.30
Injury and poisoning (800–999)	43118	65.84
Symptoms, signs and ill-defined conditions (780–799)	5234	7.99
Nervous system (320–389)	3385	5.12
Digestive system (520–579)	2276	3.48
Respiratory system (460–519)	2000	3.05
Genitourinary system (580–629)	822	1.26
Circulatory system (390–459)	454	0.69
Skin and subcutaneous tissue (680–709)	443	0.68
Endocrine, nutritional and metabolic disease and immunity disorder (240–279)	397	0.61
Mental disorder (290–319)	330	0.50
Congenital anomalies (740–759)	234	0.36
Neoplasms (140–239)	171	0.26
Malignant (140–208)	123	0.19
Benign (210–229)	48	0.07
Infectious and parasitic disease (001–139)	111	0.17
Blood and blood-forming organs (280–289)	54	0.08
Complications of pregnancy, childbirth and the puerperium (630–676)	10	0.02

## Discussion

To the best of our knowledge, the current nationwide population-based study is the first to prove that acupuncture decreased the risk of stroke in patients with fibromyalgia. In this study, we found that the benefits of acupuncture for reducing the incidence of stroke in patients with fibromyalgia were independent of sex, age, baseline comorbidities and drug use. A strength of this study is the use of a comprehensive, large-scale database. Taiwan’s NHIRD provides a tremendous sample size with long-term follow-up database, which can reduce the bias of selection and participation [18].

The prothrombotic state, chronic inflammation, oxidative stress, comorbidities, and conventional treatment might increase the risk of stroke in patients with fibromyalgia. Patients with fibromyalgia had a higher level of fibrinogen, platelet count, and platelet distribution width, and this prothrombotic state might increase the risk of thrombosis-related stroke in patients with fibromyalgia [19, 20]. Patients with fibromyalgia may had chronic pain, emotional distress and impaired daily activities. Emotional distress and physical inactivity have been considered as risk factors of stroke [21]. Management of psychosocial stress and increasing physical activity were recommended in preventing stroke [22, 23]. One to two times of moderate to vigorous intensity physical activity per week might prevent stroke in general population [24]. Pre-stroke physical activity was associated with less severe stroke [25]. Chronic pain was also associated with a higher risk of cardiovascular disorders, and it may be caused by an increasing incidence of hypertension, metabolic syndrome, and obesity [26–28]. Patients with depression and sleep disorders, which were common comorbidities of fibromyalgia, had a higher risk of stroke, and the possible mechanisms included chronic inflammation, oxidative stress, and atherosclerosis [29–33]. These findings suggested that improvement of the emotional distress, increasing activities, reliving chronic pain, and improvement of sleep quality may have beneficial effect on stroke prevention.

The Food and Drug Administration (FDA) in the USA has approved pregabalin, duloxetine, and milnacipran as treatments for fibromyalgia [3, 5]. In elderly people without cardiovascular diseases, consumption of gabapentin or pregabalin for 3 months was associated with an increased risk of atrial fibrillation, which is a risk factor for ischemic stroke [34]. NSAIDs and opioids are frequently used for pain control in patients with fibromyalgia. Previous studies revealed that NSAIDs were associated with an increased risk of ischemic stroke [35] and hemorrhagic stroke [36]. Long-term use of opioid could induce oxidative stress to activate neuro-immune system and cause stroke [37]. It’s important to prevent stroke in patients who received the conventional treatments of fibromyalgia.

The possible effects of acupuncture in reducing the risk of stroke may be through improvement of prothrombotic state, inhibition of inflammation, and modulation of endocannabinoid system. Electroacupuncture decreased plasma fibrinogen level in an acute mental stress rat model [38], and it could decrease circulating fibrinogen as well as tissue plasminogen activator in women with polycystic ovary syndrome [39]. Whether acupuncture can reduce plasma fibrinogen in patients with fibromyalgia needs to be evaluated. We have conducted a double-blinded, randomized control trial to investigate the effectiveness of acupuncture on patients with fibromyalgia, where plasma fibromyalgia level, platelet count, and platelet distribution width can be evaluated (ClinivslTrial.gov identifier: NCT02583334). Inflammation plays an important role in the pathophysiology of atherosclerotic plaque destabilization and thromboembolic events such as stroke [40, 41]. Electroacupuncture pretreatment exerted anti-inflammatory effects with activation of α7 nicotinic acetylcholine receptors (α7nAChR) and protected the brain from transient cerebral ischemic injury in rats [42]. Pretreatment of electroacupuncture suppressed extracellular signal regulated-kinase 1/2 (ERK1/2), and activated signal transducer and activator of transcription 3 (STAT3), phosphorylation of glycogen synthase kinase-3β (GSK-3β), and epsilon protein kinase C mediating anti-apoptosis mechanism to prevent the occurrence of focal cerebral ischemia through the cannabinoid receptor type 1 (CB1) receptors [43–47].

Previous clinical trials showed that acupuncture could relieve pain, reduce anxiety, increase physical activity, and improve quality of life in patients with fibromyalgia [11–17], and these effects might reduce the incidence of stroke in patients with fibromyalgia. Anti-nociceptive effect, inhibition of serotonin, and anti-inflammation were the proposed mechanisms of acupuncture for fibromyalgia treatment. Mechanical stimulation such as insertion and rotation of needles triggered the increase of extracellular adenosine degrading from ATP in response to local stimuli [48]. Acupuncture also up-regulated endogenous opiates including dynorphin, endorphin, and encephalin in plasma and cerebrospinal fluid to relieve pain [49]. In addition, electroacupuncture at the PC6 acupoint of mice increased orexin A and decreased GABA levels in the ventrolateral periaqueductal gray in response to acute thermal nociceptive stimuli and mechanical allodynia [50].

Transient receptor potential vanilloid 1 (TRPV1) is known to play a crucial role in neuropathic and inflammatory pain. Electroacupuncture inhibited the hyper-excitable of the dorsal root ganglion neurons and expression of TRPV1 and TRPV4 in the spinal cord of a murine model of fibromyalgia [51]. TRPV1 was also found to be involved in ischemic stroke and neurological deficits in a murine model of middle cerebral artery occlusion [52]. Pretreatment with electroacupuncture had a neuroprotective effect in rats with cerebral ischemia-reperfusion injury through reducing the infarct volumes, decreasing the risk of oxidative stress injury, inhibiting inflammatory cytokine production, and suppressing the expression of TRPV1 [53].

Hyperalgesia and allodynia can lead to central sensitization in fibromyalgia patients by activating the NMDAR in the spinal cord and brain sensory transmitting pathways [54, 55]. Acupuncture inhibited NMDAR signaling in the dorsal root ganglion in murine model of fibromyalgia [56]. Induction of cell necrosis or apoptosis by over activation of NMDARs might cause ischemic brain injury, and NMDAR antagonists were found to protect neurons from ischemic death in a model of middle cerebral artery occlusion [57]. The effectiveness of acupuncture in preventing stroke might be associated with neural protection via the suppression of the NMDAR signaling cascade.

A previous clinical trial suggested that the effectiveness of acupuncture in treating fibromyalgia might result from changes in the serum serotonin and substance P levels [58]. Acupuncture was shown to modulate the serotonin system in the dorsal raphe nuclei via the regulation of the serotonin transporter and local tissue via the degranulation of mast cells in murine models [59, 60]. Elevated plasma serotonin levels leading to platelet aggregation have also been observed in individuals with cardiovascular diseases, including hypertension, atherosclerosis, coronary artery disease, and arterial thrombosis [61]. The modulating effect of acupuncture on serotonin secretion and reuptake may be a possible explanation for its effectiveness in stroke prevention.

Elevation of IL-1β, IL-6 and IL-8, and mast cells mediating microglia activation through the production of proinflammatory cytokines were found in patients with fibromyalgia [62–64]. Electroacupuncture suppressed microglia-induced IL-1β and IL-18 expression in rats [65], and it also suppressed the expression of IL-1*β* mRNA and TNF-*α* mRNA in the spinal cord in rats [66]. We also found that acupuncture treatment could decrease inflammatory cytokines in patients with fibromyalgia in the preliminary data of our clinical trial (ClinivslTrial.gov identifier: NCT02583334).

There were some limitations in our study. First, detailed information about the severity of fibromyalgia and prothrombotic status, including pain scores, the severity of widespread pain, impairment in daily activities, and fibrinogen level were not provided in the NHIRD. Patients with fibromyalgia of different severities and its comorbidities may undergo corresponding treatments; thus, we performed propensity score matching with a ratio of 1:1 to minimize the effects of confounding factors on our results in this study. In the acupuncture and non-acupuncture groups, we found that there was no difference in the percentages of patients who used NSAIDs and oral steroids. The second limitation is that lifestyle information, such as exercise, smoking, and body mass index (BMI), were not available in this database. We could only use the ICD-9-CM code to identify the patients with alcoholism, tobacco dependence, and obesity, therefore, rates of smoking and alcohol consumption in the current study might be underestimated. Third, the NHIRD did not provide complete details about acupuncture treatment protocols, including acupoint selection, manipulation skills and retention period of needle. TCM doctors selected different acupoints and manipulated the needles for each patient according to TCM theory, depending on the patterns of clinical manifestations that could change during disease progression. Due to the regulations of the NHI program, the maximal times of acupuncture visits per month is 15, so visit times of acupuncture reported in our study might be underestimated. Some patients may undergo additional acupuncture treatment by self-pay, and these visits are not recorded in the NHIRD database. Detail information of patients with fibromyalgia, prothrombotic status, and acupoint selection can be provided by the clinical trial, and it’s the reason that we conducted a double-blinded, randomized control trial to evaluate the effectiveness of acupuncture on fibromyalgia (NCT02583334). However, it’s difficult to design a clinical trial to evaluate whether acupuncture can prevent stroke in patients with fibromyalgia because long-term follow-up is need. This retrospective cohort study used a large-scale database with long-term follow-up in Taiwan could provide the “real-world evidence”. More clinical and basic science studies need to be conducted to provide more evidence and elucidate the mechanism of fibromyalgia.

## Conclusions

Our study found that acupuncture might have a beneficial effect on reducing the risk of stroke in patients with fibromyalgia in Taiwan. This significant finding can be used to guide additional clinical and basic science studies.
