# Correction: Acupuncture decreased the risk of stroke among patients with fibromyalgia in Taiwan: A nationwide matched cohort study

**DOI:** 10.1371/journal.pone.0314221

**Published:** 2024-11-14

**Authors:** Ming-Cheng Huang, Hung-Rong Yen, Cheng-Li Lin, Yu-Chen Lee, Mao-Feng Sun, Mei-Yao Wu

Fig 1 is uploaded incorrectly. Please see the correct Fig 1 here.

**Fig 1:**
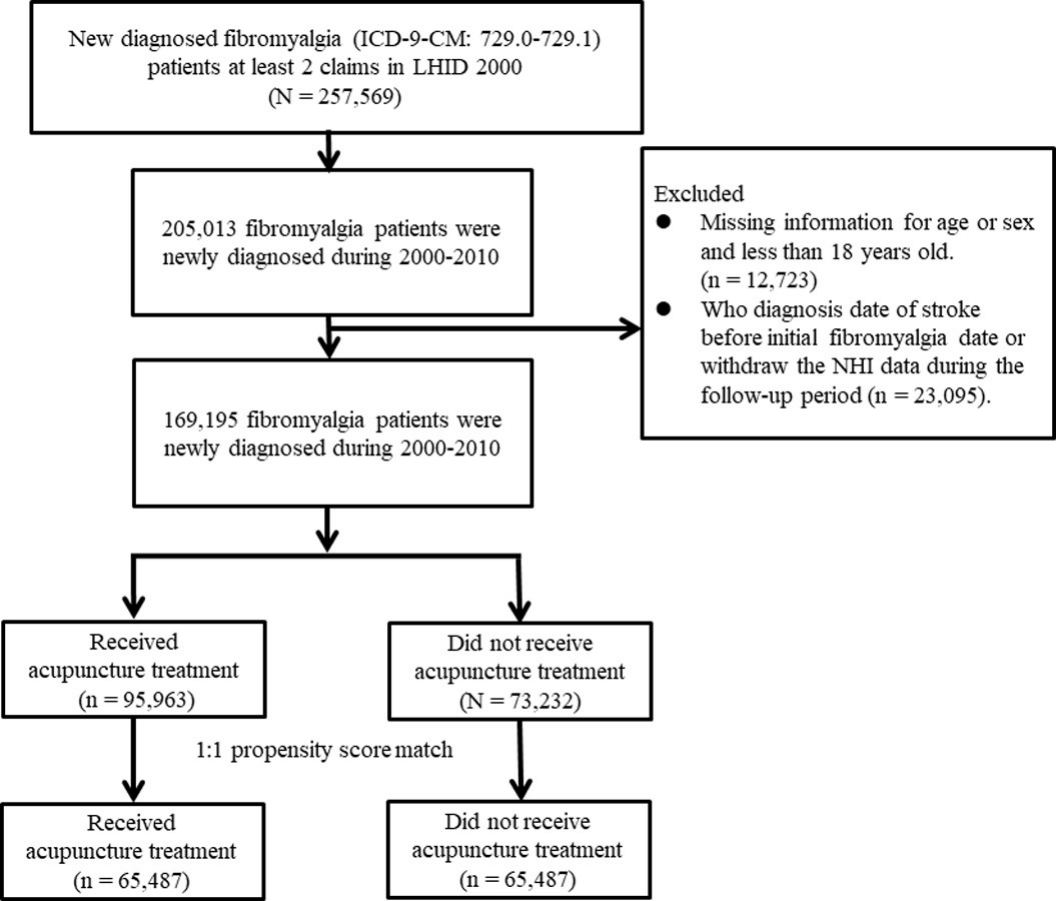
Study population flowchart. We identified 169,195 patients who were newly diagnosed with fibromyalgia between 2000 and 2010. After 1:1 propensity score matching by sex, age, comorbidities, drug use, diagnostic year and index year was performed, the acupuncture cohort and non-acupuncture cohort both comprised 65,487 patients.
